# Pterostilbene Combating Cyclophosphamide-Induced Central Neuro-Inflammation in Rats: Effect on GFAP, PPAR- γ, NLRP3, COX-2 and Nrf2/HO-1 Pathway

**DOI:** 10.3390/biomedicines14081779

**Published:** 2026-08-07

**Authors:** Rania F. Ahmed, Hadir Farouk, Yosra A. Hussien, Marwa E. Shabana, Maha Nasr

**Affiliations:** 1 Department of Pharmacology, Medical Research and Clinical Studies Institute, National Research Centre, Dokki, Giza 12622, Egypt; 2 Department of Pathology, Medical Research and Clinical Studies Institute, National Research Centre, Dokki, Giza 12622, Egypt; 3 Department of Pharmacology and Therapeutics, College of Medicine and Health Sciences, United Arab Emirates University, Al Ain 15551, United Arab Emirates; 4 Department of Pharmaceutics and Industrial Pharmacy, Faculty of Pharmacy, Ain Shams University, Cairo 11566, Egypt

**Keywords:** cyclophosphamide, pterostilbene, neurotoxicity, brain injury, oxidative stress, inflammation

## Abstract

**Background/Objectives**: The clinical efficacy of cyclophosphamide, a commonly used chemotherapeutic drug, is often restricted by off-target neurotoxicity, which is primarily caused by oxidative stress, neuroinflammation, and apoptosis. With the aim of finding natural neuroprotective agents, pterostilbene was investigated in this study, either in conventional form or when solubilized as a nanoemulsion, for its neuroprotective potential against cyclophosphamide-induced neurotoxicity. **Methods**: Female Wistar rats were orally administered pterostilbene, either in its conventional form (50 mg/kg p.o) or nanoemulsion form (25 and 50 mg/kg p.o), for 15 days. Cyclophosphamide was injected once on day 14 (200 mg/kg ip). **Results:** Our findings revealed that administering pterostilbene as a nanoemulsion or in its conventional powder form managed to counteract the cyclophosphamide-induced intoxication features via disrupting the oxidative stress–inflammasome–cytokine axis; by upregulating cortical and hippocampal PPAR-γ, Nrf2, HO-1, BCL2 levels and the BCL2/Bax ratio; and downregulating NLRP3, IL-1β, COX-2, Bax and GFAP levels. Further histopathological and immunohistochemical evaluation confirmed the improved neuronal morphology, reduced degeneration and apoptosis, restoration of cortical and hippocampal architecture, and normalization of GFAP expression levels, approaching those of the control group upon treatment with either conventional or nanoformulated pterostilbene. **Conclusions**: The promising nature of the nanoemulsion formulation, particularly when administered at the high dose level, was evidenced, hence emphasizing the important role of proper formulation of natural bioactives such as pterostilbene in maximizing their therapeutic potential.

## 1. Introduction

The mustard-alkylating agent cyclophosphamide is a member of the oxazaphosphorine family [1]. Being one of the most significant cytotoxic alkylating medications, it is frequently prescribed for patients with leukemia, ovarian cancer, breast cancer, small cell lung cancer, multiple myeloma, lymphoma, neuroblastoma, or sarcoma [2,3,4]. Unfortunately, one of the main drawbacks of cyclophosphamide is the induction of neurotoxicity as a result of disrupting the brain’s oxidative balance and the induction of an oxidative inflammatory cascade, resulting in extensive cellular degeneration, pro-inflammatory response, and apoptosis [5,6,7,8].

Many plant species, such as crops utilized in human diets and plants used in traditional medicine, produce stilbenes, which include pterostilbene (3′,5′-dimethoxy-resveratrol), a methoxylated derivative of resveratrol (3,5,4′-trihydroxystilbene) [9]. Due to its several biological functions, including neuroprotective, anti-inflammatory, antioxidant, antiapoptotic and metabolic regulatory properties, in addition to its low toxicity and relatively few side effects, pterostilbene has attracted a lot of attention for its use in the protection from several medical conditions, including neurological disorders [10,11,12,13,14]. Because the methoxy group is substituted for the hydroxyl group, pterostilbene, a structural analog of resveratrol, exhibits greater blood–brain barrier (BBB) permeability as a result of its increased lipid solubility [15,16]. Additionally, pterostilbene demonstrates antioxidant response-induced neuroprotection mediated by Nrf2 (nuclear erythroid-related factor) [17]. Therefore, the purpose of this study was to investigate pterostilbene’s underlying mechanism in cyclophosphamide-induced neurotoxicity by focusing on oxidative stress, inflammatory reactions, and apoptotic pathways. Pterostilbene is known to exhibit low water solubility, which hinders the realization of its full therapeutic potential [18]. Its formulation in oil-in-water (o/w) nanoemulsion was attempted in the current study to permit its full solubilization, hence maximizing its neuroprotective efficacy in combating cyclophosphamide-induced brain injury in rats. A comparative study between the effect of pterostilbene administered as conventional powder versus its administration in nanoemulsion form was also conducted in the current study.

## 2. Materials and Methods

### 2.1. Materials

Pterostilbene was obtained from the Skinactives company, Gilbert, Arizona, AZ, USA. All other chemicals were purchased from Sigma Aldrich company, Darmstadt, Germany.

### 2.2. Preparation of Pterostilbene O/W Nanoemulsions

Pterostilbene nanoemulsions were prepared using the spontaneous emulsification technique. In brief, isopropyl myristate and Tween 80 (6.5% *v*/*v* each) were used to dissolve pterostilbene. This mixture was then slowly introduced into deionized water under continuous magnetic stirring at room temperature [19]. Two nanoemulsion formulations containing pterostilbene at two concentrations were created, 2.5 mg/mL and 5 mg/mL, which corresponded to administered dosages of 25 and 50 mg/kg, respectively. The total dosing volume was roughly 2.0–2.1 mL per rat depending on body weight. The nanoemulsions were characterized for their particle size, polydispersity, zeta potential when freshly prepared and upon storage in refrigerated conditions (2–8 °C) for three months (Malvern Zetasizer, NanoZS, Malvern, UK). The morphology of the nanoemulsion 50 mg/kg was visualized using transmission electron microscopy (Jeol, JEM 1010, Tokyo, Japan).

### 2.3. Animals

Wistar albino adult female rats (7 weeks of age, 190–200 gm) were purchased from the animal housing colony of the National Research Center (NRC). Before beginning the experimental study, the animals (8 weeks old, 200–210 g) were allowed to acclimate for seven days upon arrival. In accordance with Egyptian regulations and in accordance with the ARRIVE guidelines, the NRC Medical Research Ethics Committee approved all experimental protocols (the study is a part of a general study on the effect of pterostilbene against cyclophosphamide-induced toxicity in female rats with approval No. 01430525).

### 2.4. In Vivo Study

Six groups of rats were randomly selected (8 rats each) as follows: G1; normal control receiving only daily oral ingestions of distilled water 15 days (depending on body weight, roughly 2–2.1 mL/rat) [20,21]. G2; cyclophosphamide control, which received daily oral ingestions of distilled water only for 15 days (depending on body weight, roughly 2–2.1 mL/rat). G3; vehicle control NV, which received daily oral ingestions of the nano-emulsion vehicle (unloaded with pterostilbene) for 15 days (depending on body weight, roughly 2–2.1 mL/rat). G4, G5 and G6; test groups: three groups which received pterostilbene orally for 15 days and were divided into two Pterostilbene nano-formula groups, receiving two different dose levels of the nano-formula PTNL and PTNH (25 and 50 mg/kg p.o) (depending on body weight, roughly 2–2.1 mL/rat). Pterostilbene powder group PTPF: receiving pterostilbene powder (50 mg/kg p.o) [22], where the powder suspension was freshly prepared in distilled water so that each rat would receive about 2–2.1 mL of the final suspension depending on body weight. Every group, with the exception of the normal control, was injected with cyclophosphamide once on day 14 (200 mg/kg ip) [23,24]. On day 16, 48 h after cyclophosphamide injection [25,26], decapitation was used to sacrifice rats. Brain samples were collected and either stored in 10% formalin for additional histological investigations or stored below −80 °C for additional biochemical analyses.

### 2.5. Parameters Measured

#### 2.5.1. ELISA Parameters

Following the provided manual, ELISA kits from SinoGeneClon Biotech Co. Ltd, Hangzhou, China. were used to assess brain Nrf2 (Cat. No. SG-20003), COX-2 (Cat. No. SG-20355), HO-1 (Cat. No. SG-20658), PPAR- γ (Cat. No. SG-20819), NLRP3 (Cat. No. SG-24214), IL-1β (Cat. No. SG-20260), Bax (Cat. No. SG-20676), and BCL2 (Cat. No. SG-20401), were assessed using following the homogenization of brain tissues in saline (after separation of brain regions homogenization in saline to generate 20% homogenate was performed after that centrifugation and separation of the supernatant was done, which was used in the ELISA assessments.

#### 2.5.2. Histopathological H&E Examination

After sacrifice, specimens were autopsied from the brain tissues of distinct groups, preserved in neutral buffered formalin (10%) for 24 h, washed properly under flowing water, dried in ascending grades of alcohol, fixed with paraffin wax after being washed with xylene. Serial sections of 4 μm thick were cut and stained with H&E for histopathological investigation [27]. Finally, pictures were reviewed and captured using a digital camera (Microscope Digital Camera DP 70, Leica, Tokyo, Japan) and processed with Adobe Photoshop, Version 8.0.

#### 2.5.3. Immunohistochemistry

Using mouse monoclonal antibodies (catalog no. sc-33673; Santa Cruz Biotechnology, Dallas, TX, USA) and the Leica Bond polymer refine detection kit (catalog no. DS9800; Novocastra, Nussloch, Germany), the highly specific glial cell marker glial fibrillary acidic protein (GFAP) was identified using the immunohistochemical method in accordance with the manufacturer’s protocol. Immunohistochemical stains with appropriate positive and negative controls were blindly evaluated and placed into one of two categories: positive or negative [28]. Morphometric analysis of the region where the brown-colored GFAP is located was carried out at ×100 magnification using a light microscope, Olympus CX-41, with DP 12 Olympus digital camera (Olympus Optical Co. Ltd., Tokyo, Japan). The interactive software of the system determines the area to be measured as an area per field in micrometers squared, area fraction, and area percentage. The total, mean, SD, SE, minimum area, and maximum area measured are shown automatically on the monitor as a table. Eight field measurements were taken in total (4 fields per slide); two slides for each group were examined. The examiner of the slides was blind to the groups and was given slides in numeric codes to avoid bias. Area per field was calculated in micrometers squared for every field, and the mean for eight fields was calculated for each group and represented as area percent on the graph [29].

### 2.6. Statistical Analysis

The Shapiro–Wilk and/or Anderson-Darling and Brown-Forsythe tests confirmed normality and homogeneity of variances, respectively. Differences among groups were assessed by one-way analysis of variance (ANOVA), with Tukey–Kramer’s post hoc test used for multiple comparisons. The results were then expressed as means ± S.E. For the biochemical assays, each group employed eight samples (n = 8). Regarding the morphometric measures, two slides were used for each group, and a total of eight field measurements were made (four per slide). The displayed graphs were created using the GraphPad Prism application (version 10, CA, San Diego, USA). Asterisks were used to indicate the significance in the graph plot (*p* value ˂ 0.05).

## 3. Results

### 3.1. Characterization of the Nanoemulsions

The nanoemulsions were successfully prepared after a preliminary study delineating the solubility of pterostilbene in isopropyl myristate and Tween 80 of 130 and 15 mg/mL, respectively. The low- and high-dose nanoemulsions displayed particle sizes of 180.40 ± 0.56 and 195.28 nm, polydispersity of 0.31 ± 0.15 and 0.34 ± 0.11, and zeta potential of −12.14 ± 2.85 and −11.37 ± 1.25 mV, respectively, with no statistically significant changes upon storage for 3 months (*p* > 0.05). The nanoemulsion displayed a spherical morphology, as shown in Supplementary Figure S1.

### 3.2. Effect on Cortical and Hippocampal Nrf2 Levels

**Figure 1:**
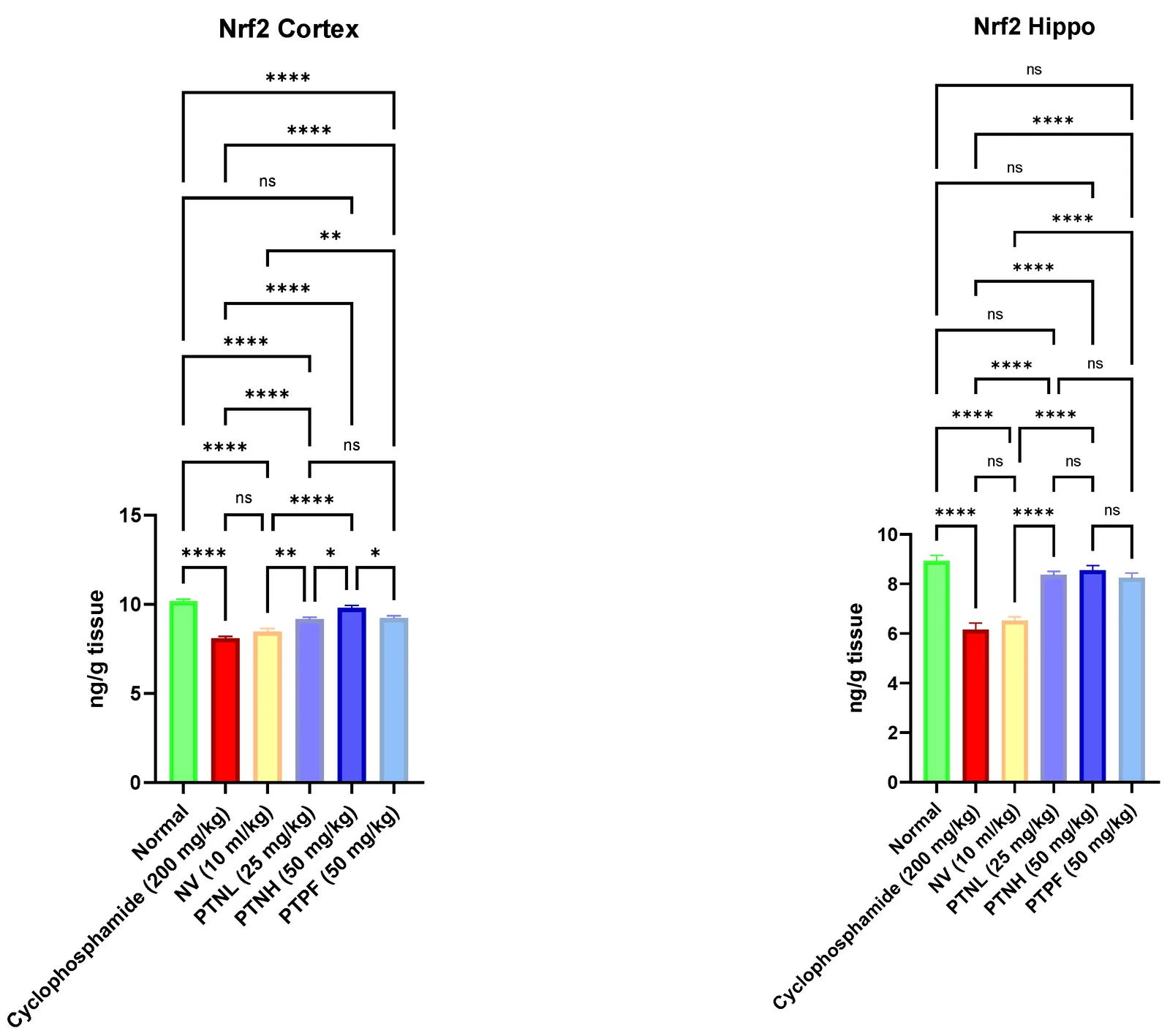
Effect of pterostilbene administration in powder and nanoemulsion forms on the cortical and hippocampal Nrf2 levels. Differences among groups were assessed by one-way analysis of variance (ANOVA), with Tukey–Kramer’s post hoc test. Results represent mean ± S.E (n = 8). Asterisks *,**,**** represent degree of significant difference, ns: non significant. NV: vehicle control; PTNL: low-dose pterostibene nanoemulsion group; PTNH: high-dose pterostibene nanoemulsion group; PTPF: Pterostilbene freshly prepared powder suspension group.

As represented in Figure 1**,** when compared to the normal control, cyclophosphamide showed a significant decline in the cortical and hippocampal Nrf2 levels (*p* < 0.0001). Additionally, a non-significant difference was detected between the group that ingested the unloaded nano-vehicle NV and the cyclophosphamide control. On the contrary, the group ingesting the lower dose of the nanoemulsion PTNL (25 mg/kg) and that ingesting pterostilbene in its suspended conventional form PTPF (50 mg/kg) elevated the cortical Nrf2 level significantly compared to the cyclophosphamide control (*p* < 0.0001), while the high dose level of the nanoemulsion PTNH (50 mg/kg) normalized the Nrf2 cortical level. In addition, all groups, PTNL, PTNH and PTPF, normalized the hippocampal Nrf2 level.

### 3.3. Effect on Cortical and Hippocampal HO-1 Levels

**Figure 2:**
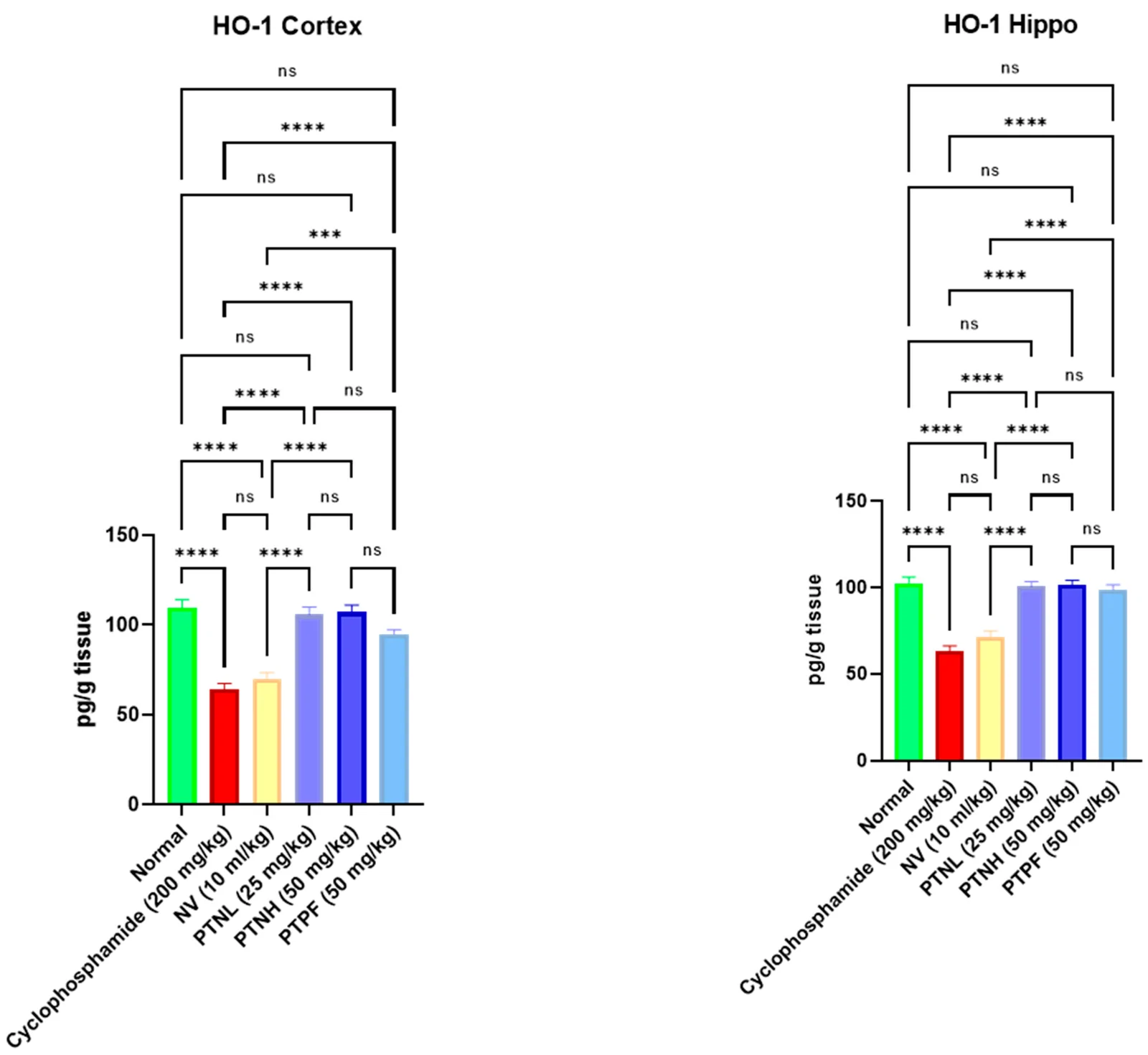
Effect of pterostilbene administration in powder and nanoemulsion forms on the cortical and hippocampal HO-1 levels. Differences among groups were assessed by one-way analysis of variance (ANOVA), with Tukey–Kramer’s post hoc test. Results represent mean ± S.E (n = 8). Asterisks ***, **** represent degree of significant difference, ns: non significant. NV: vehicle control; PTNL: low-dose pterostibene nanoemulsion group; PTNH: high-dose pterostibene nanoemulsion group; PTPF: pterostilbene freshly prepared powder suspension group.

As revealed in Figure 2, when compared to normal control, cyclophosphamide resulted in a significant decline in the cortical as well as the hippocampal HO-1 levels (*p* < 0.0001), while a non-significant difference was found between the unloaded nano-vehicle NV group and the cyclophosphamide control. Conversely, all treatment groups, PTNL, PTNH and PTPF, normalized the HO-1 cortical and hippocampal levels.

### 3.4. Effect on Cortical and Hippocampal COX-2 Levels

**Figure 3:**
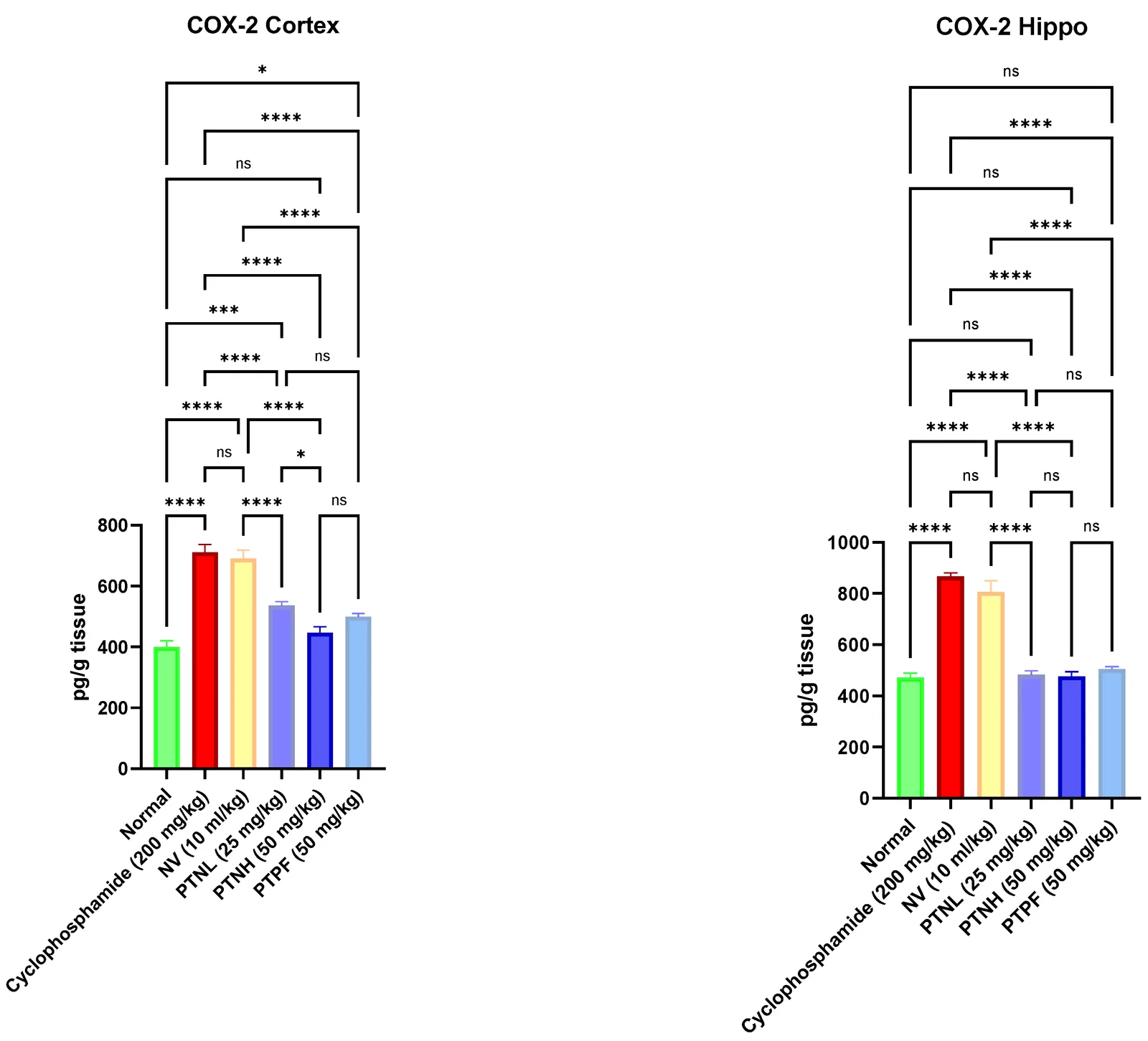
Effect of pterostilbene administration in powder and nanoemulsion forms on the cortical and hippocampal COX-2 levels. Differences among groups were assessed by one-way analysis of variance (ANOVA), with Tukey–Kramer’s post hoc test. Results represent mean ± S.E (n = 8). Asterisks *, ***, **** represent degree of significant difference, ns: non significant. NV: vehicle control; PTNL: low-dose pterostibene nanoemulsion group; PTNH: high-dose pterostibene nanoemulsion group; PTPF: Pterostilbene freshly prepared powder suspension group.

As shown in Figure 3, cyclophosphamide resulted in a significant elevation in the cortical as well as the hippocampal COX-2 levels as compared to normal control (*p* < 0.0001), with no significant change detected among the group ingesting the unloaded nano-vehicle NV and the cyclophosphamide control. Conversely, both the group ingesting the lower dose of the nanoemulsion PTNL (25 mg/kg) and that ingesting pterostilbene in the suspended conventional form PTPF (50 mg/kg) significantly elevated the cortical COX-2 level as compared to the cyclophosphamide control (*p* < 0.0001), while nanoemulsion PTNH (50 mg/kg) in the high dose level normalized the COX-2 cortical level. On the other hand, all groups, PTNL, PTNH and PTPF, normalized the hippocampal COX-2 level.

### 3.5. Effect on Cortical and Hippocampal NLRP3 Levels

**Figure 4:**
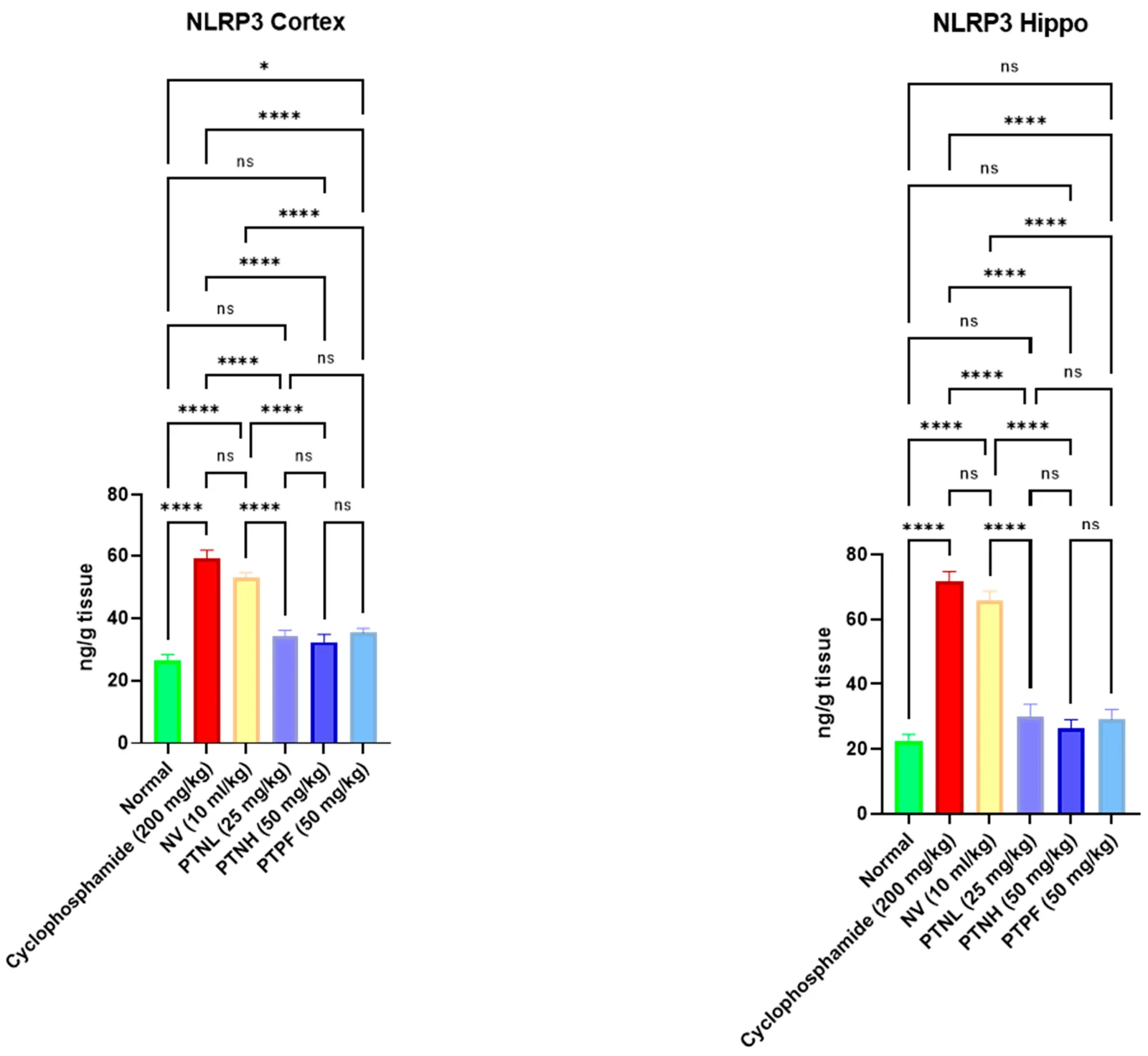
Effect of pterostilbene administration in powder and nanoemulsion forms on the cortical and hippocampal NLRP3 levels. Differences among groups were assessed by one-way analysis of variance (ANOVA), with Tukey–Kramer’s post hoc test. Results represent mean ± S.E (n = 8). Asterisks *, **** represent degree of significant difference, ns: non significant. NV: vehicle control; PTNL: low-dose pterostibene nanoemulsion group; PTNH: high-dose pterostibene nanoemulsion group; PTPF: pterostilbene freshly prepared powder suspension group.

As shown in Figure 4, when compared to the normal control, cyclophosphamide resulted in a significant increase in the cortical and hippocampal NLRP3 levels (*p* < 0.0001), while a non-significant difference was detected between the unloaded nano-vehicle NV group and the cyclophosphamide control. Conversely, the group that ingested pterostilbene in the suspended conventional form PTPF (50 mg/kg) resulted in a significant reduction in the cortical NLRP3 level when compared to the cyclophosphamide control (*p* < 0.0001); however, both groups ingesting the nanoemulsions resulted in normalizing NLRP3 cortical levels. Additionally, all treatment groups normalized hippocampal NLRP3 levels.

### 3.6. Effect on Cortical and Hippocampal IL-1β Levels

**Figure 5:**
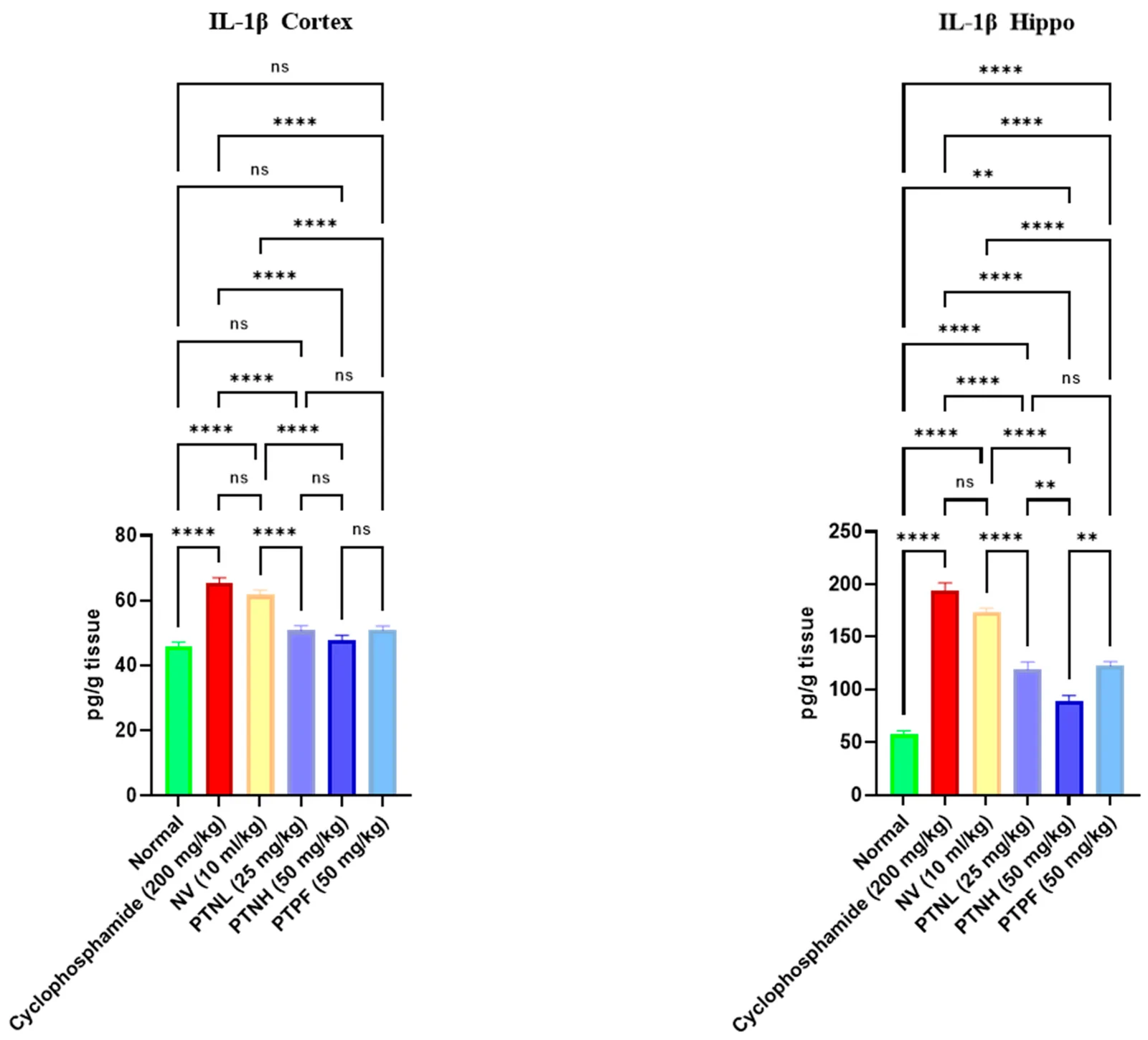
Effect of pterostilbene administration in powder and nanoemulsion forms on the cortical and hippocampal IL-1β levels. Differences among groups were assessed by one-way analysis of variance (ANOVA), with Tukey–Kramer’s post hoc test. Results represent mean ± S.E (n = 8). Asterisks **, **** represent degree of significant difference, ns: non significant. NV: vehicle control; PTNL: low-dose pterostibene nanoemulsion group; PTNH: high-dose pterostibene nanoemulsion group; PTPF: pterostilbene freshly prepared powder suspension group.

As shown in Figure 5, cyclophosphamide resulted in a significant increase in the cortical as well as the hippocampal IL-1β levels as compared to normal control (*p* < 0.0001), and also, there was no significant difference detected between the group ingesting the unloaded nano-vehicle NV and the cyclophosphamide control. Conversely, all treatment groups normalized the cortical IL-1β level and significantly reduced the hippocampal IL-1β level as compared to the cyclophosphamide control (*p* < 0.0001).

### 3.7. Effect on Cortical and Hippocampal PPAR-γ Levels

**Figure 6:**
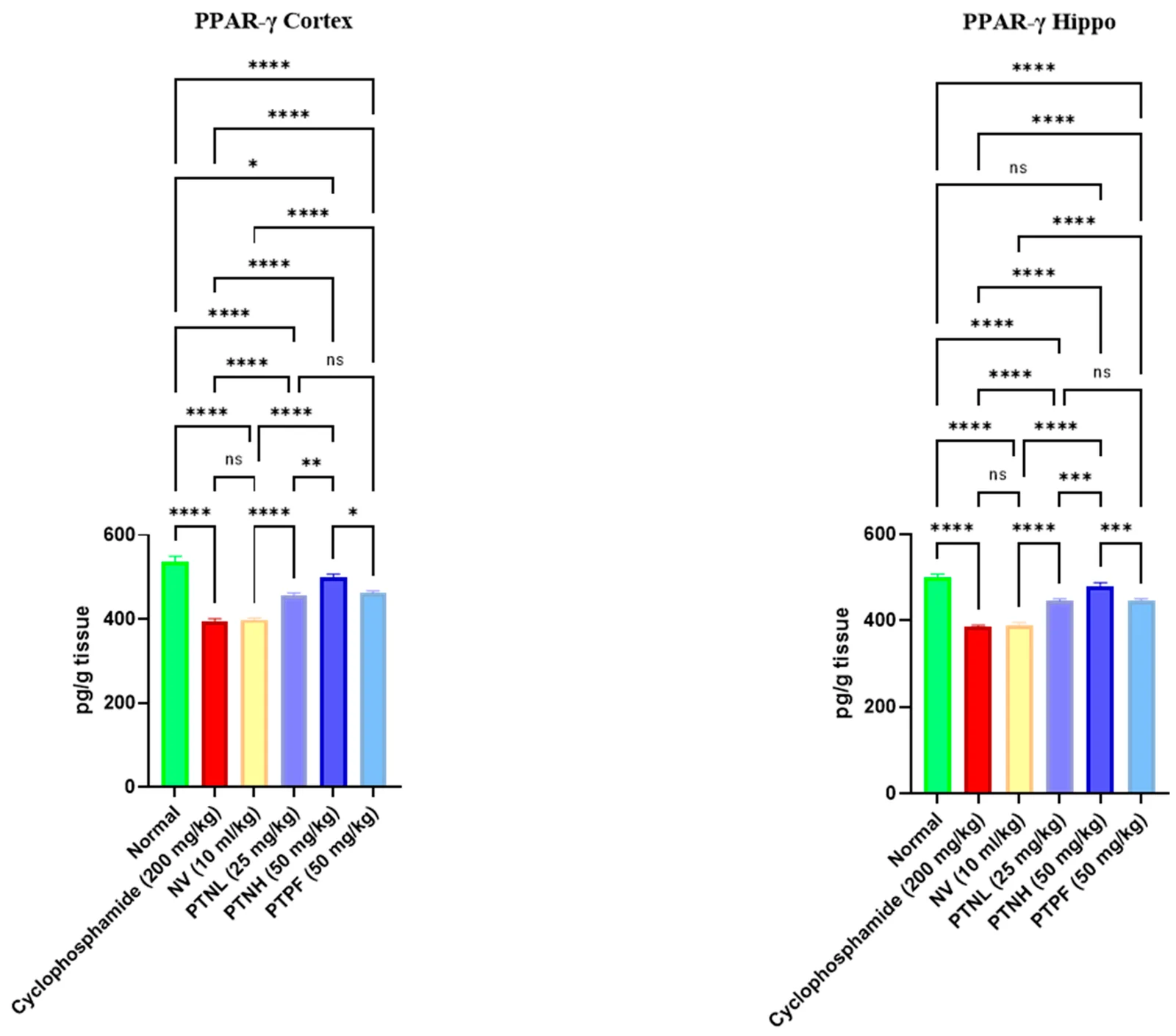
Effect of pterostilbene administration in powder and nanoemulsion forms on the cortical and hippocampal PPAR-γ levels. Differences among groups were assessed by one-way analysis of variance (ANOVA), with Tukey–Kramer’s post hoc test. Results represent mean ± S.E (n = 8). Asterisks *, **, ***, **** represent degree of significant difference, ns: non significant. NV: vehicle control; PTNL: low-dose pterostibene nanoemulsion group; PTNH: high-dose pterostibene nanoemulsion group; PTPF: pterostilbene freshly prepared powder suspension group.

As shown in Figure 6, when compared to normal control, cyclophosphamide caused a significant reduction in the cortical as well as the hippocampal PPAR-γ levels (*p* < 0.0001). Additionally, a non-significant difference was detected between the unloaded nano-vehicle NV group and the cyclophosphamide control. Meanwhile, all treatment groups significantly elevated the PPAR-γ cortical levels when compared to the cyclophosphamide control (*p* < 0.0001). In addition, both the PTNL and PTPF groups significantly increased the PPAR-γ hippocampal level when compared to the cyclophosphamide control (*p* < 0.0001). Additionally, the PPAR-γ hippocampal level was normalized in the PTNH group.

### 3.8. Effect on Cortical and Hippocampal BCL2, Bax and BCL2/Bax Levels

**Figure 7:**
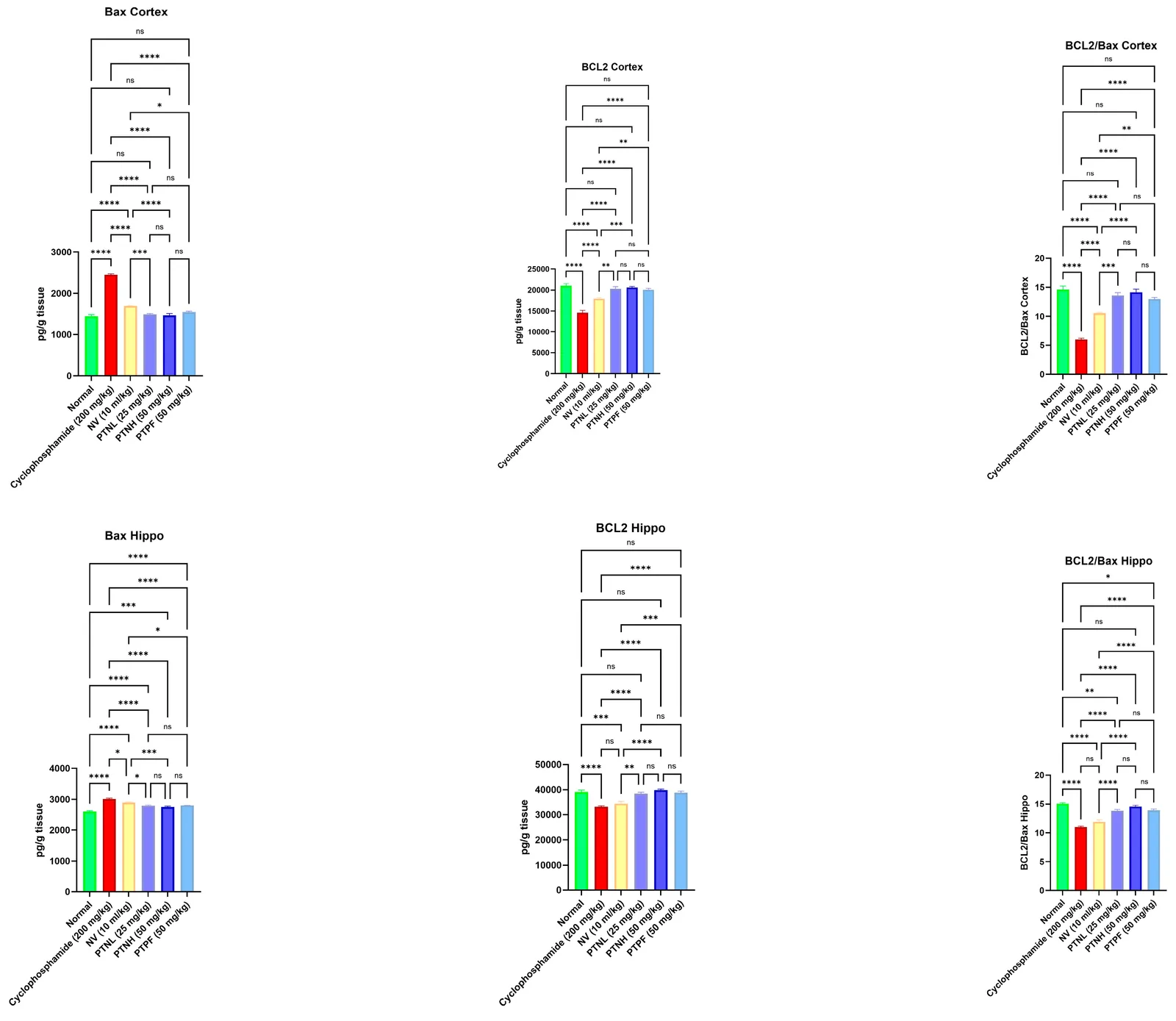
Effect of pterostilbene administration in powder and nanoemulsion forms on the cortical and hippocampal BCL2, Bax and BCL2/Bax levels. Differences among groups were assessed by one-way analysis of variance (ANOVA), with Tukey–Kramer’s post hoc test. Results represent mean ± S.E (n = 8). Asterisks *, **, ***, **** represent degree of significant difference, ns: non significant. NV: vehicle control; PTNL: low-dose pterostibene nanoemulsion group; PTNH: high-dose pterostibene nanoemulsion group; PTPF: pterostilbene freshly prepared powder suspension group.

As shown in Figure 7, cyclophosphamide resulted in a significant decline in the BCL2 level as well as the BCL2/Bax ratio, along with a marked elevation in the Bax level both in the cortex and the hippocampus as compared to normal control (*p* < 0.0001). It is worth mentioning that the group ingesting the unloaded nano-vehicle NV showed a significant decrease in the cortical and the hippocampal Bax levels when compared to the cyclophosphamide control (*p* < 0.0001 and *p* = 0.0203, respectively). Additionally, the BCL2 cortical level and BCL2/Bax cortical ratio were significantly elevated (*p* < 0.0001). Conversely, BCL2 hippocampal level and BCL2/Bax hippocampal ratio demonstrated no significance from the cyclophosphamide control. However, in contrast, all other treated groups normalized Bax cortical levels and significantly reduced its hippocampal levels when compared to the cyclophosphamide control (*p* < 0.0001). Additionally, they normalized cortical BCL2 as well as hippocampal levels and the BCL2/Bax cortical ratio. In addition, PTNH normalized the BCL2/Bax hippocampal ratio, and both PTNL as well as PTPF significantly reduced the hippocampal BCL2/Bax ratio as compared to the cyclophosphamide control (*p* < 0.0001).

### 3.9. Correlational Study Regarding the Effect of PPAR-γ on NLRP3, Nrf2 and HO-1, the Effect of COX-2 on NLRP3 and the Effect of Nrf2 on HO-1

Correlational studies (Supplementary Figure S1) revealed the presence of a negative correlation between PPAR-γ and NLPR3. Meanwhile, direct correlation between PPAR-γ and both Nrf2 and HO-1 was demonstrated. AN increased level of PPAR-γ was accompanied by a reduction in the level of NLPR3, along with an elevation in the level of Nrf2 and HO-1, both in the cortex and hippocampal areas. HO-1 and BCL2 were also found to be directly correlated to Nrf2, while Bax and NLRP3 were demonstrated to be negatively correlated to it. Similarly, NLRP3 showed a negative correlation with HO-1. Meanwhile, there was a direct correlation between COX-2 and NLRP3, as well as between NLRP3. Increased levels of Nrf2 were accompanied by an elevation in HO-1 and BCL2 along with a decrease in Bax in both brain areas under investigation. Meanwhile, elevation in either Nrf2 or HO-1 was accompanied by a reduction in NLRP3, while upregulation of COX-2 was linked to its upregulation. In addition, the increase in NLRP3 was directly correlated to the increase in IL-1β.

### 3.10. Histopathological Study and Immunohistochemistry

**Figure 8:**
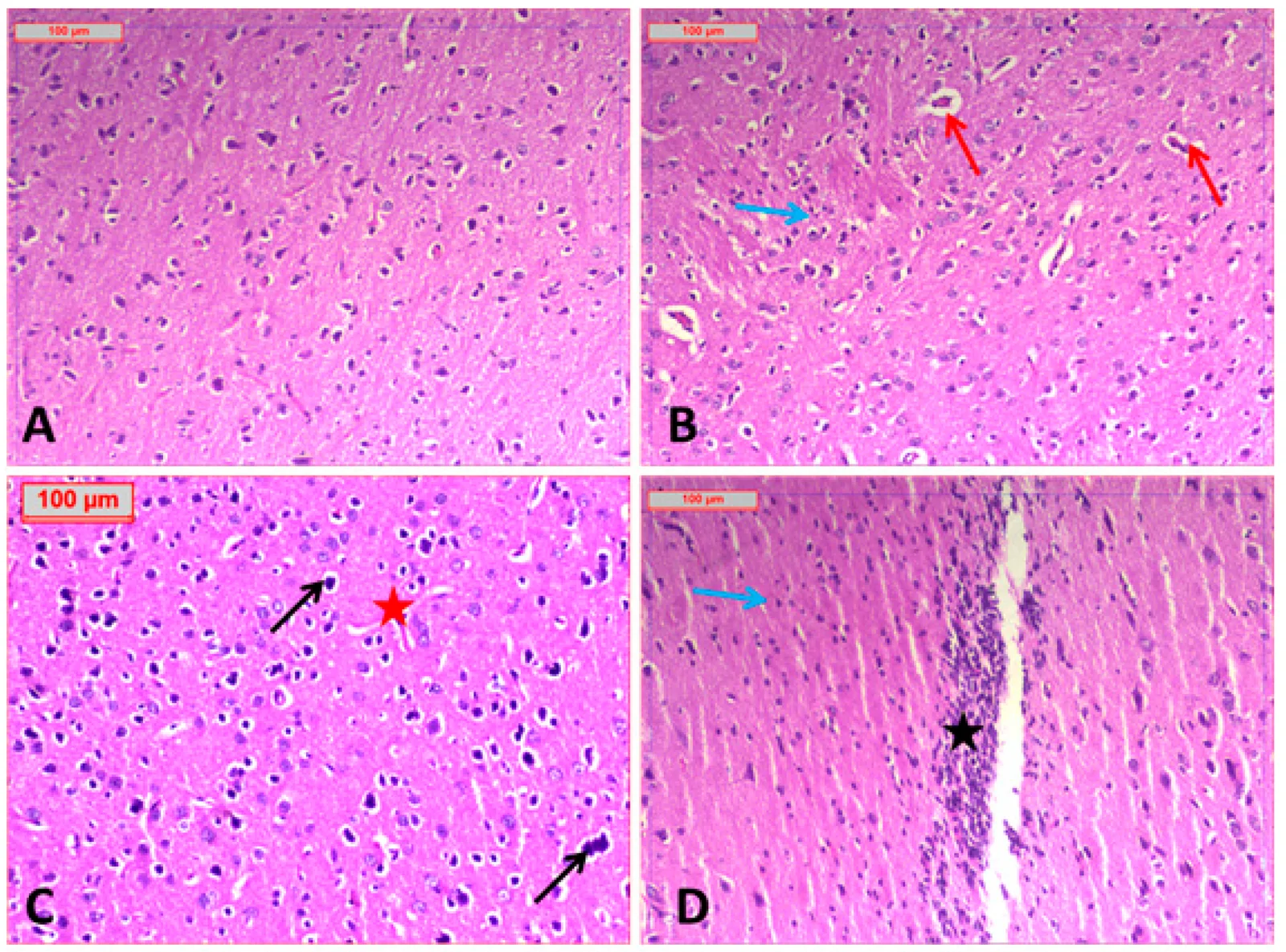
Photomicrographs of cerebral region (**A**): normal control group. (**B**–**D**): cyclophosphamide-treated group revealed degenerate and vacuolated neurocytes (black arrows), dystrophic changes, shrunken hyperchromatic neurons (red arrow), marked glial cell proliferation (black star), dilated blood vessel (red star), and scattered inflammation (blue arrow) (H&E, ×200).

**Figure 9:**
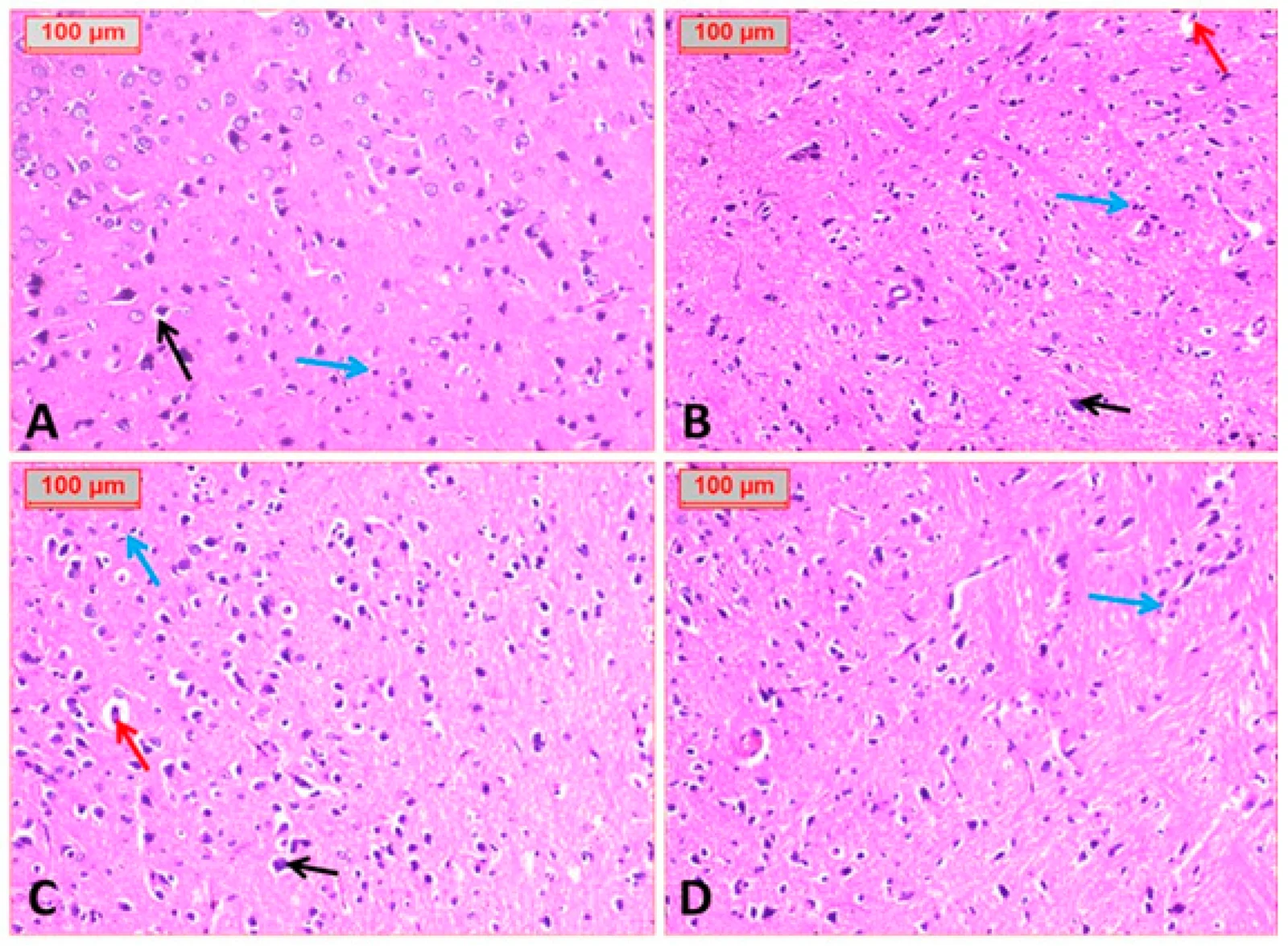
Photomicrographs of cerebral region (**A**): Vehicle group. (**B**): Powder form PTPF. (**C**): Nanoemulsion low-dose PTNL (**D**): Nanoemulsion high-dose PTNH. Black arrow: degenerated neurocyte. Blue arrow: inflammatory cells; red arrow: dystrophic neuron (H&E, ×200).

The histopathological examination of cortical sections revealed normal neuronal cells and glial cells in normal control group (Figure 8A). Cyclophosphamide-treated groups showed congested blood vessels, shrinkage, and degeneration in neurons, marked glial cell proliferation and scattered inflammatory cells (Figure 8B–D). Treatment with the vehicle form revealed increased degenerated neurons and scattered inflammatory cells (Figure 9A). Treatment with variable forms of pterostilbene revealed variable degrees of improvement as follows: Treatment with powder form revealed improvement with scattered degenerated neurocytes, shrunken neurons and moderate inflammation (Figure 9B). Treatment with low-dose nanoemulsion revealed degenerated neurocytes and shrunken neurons with mild inflammation (Figure 9C). Treatment with high-dose nanoemulsion revealed marked improvement with mild inflammation (Figure 9D).

**Figure 10:**
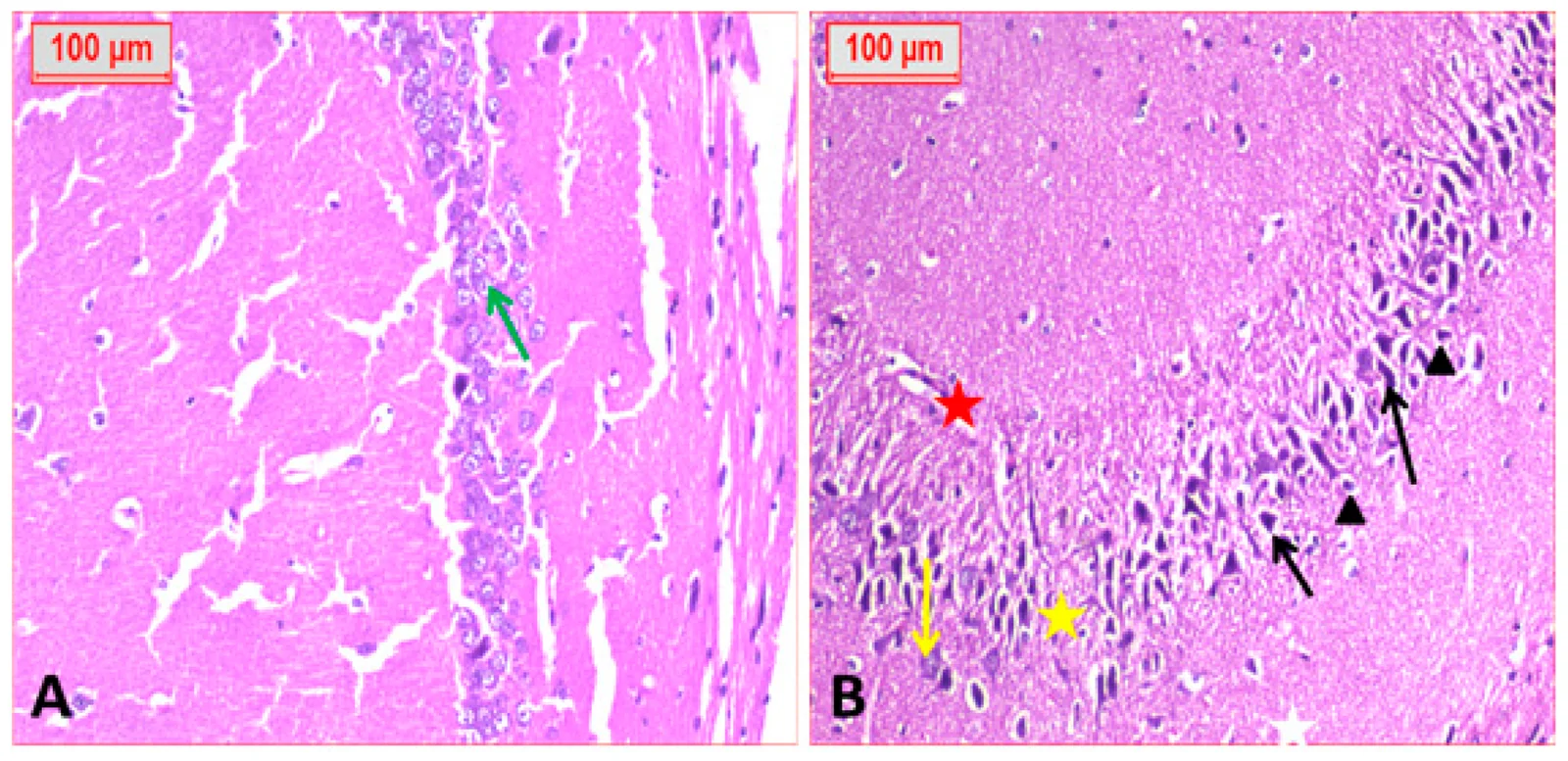
Photomicrographs of hippocampus region (**A**): normal control group with normal neurons. (**B**): cyclophosphamide-treated group with distorted and decreased thickness of pyramidal cell layer, scattered apoptotic neurons (arrow head), neurodegeneration appear as flame-like (black arrow), scattered necrotic neurons (yellow arrow), necrotic areas (yellow star), normal neurons (green arrow), and dilated blood vessels (red star) (H&E, ×200).

Histopathological examination of the hippocampus area revealed typical neurons and a normal cell layer thickness (Figure 10A). The cyclophosphamide-treated group showed distorted and reduced pyramidal cell layer thickness, scattered apoptotic neurons, neurodegeneration appearing as flame-like, scattered necrotic neuron areas and dilated blood vessels (Figure 10B).

**Figure 11:**
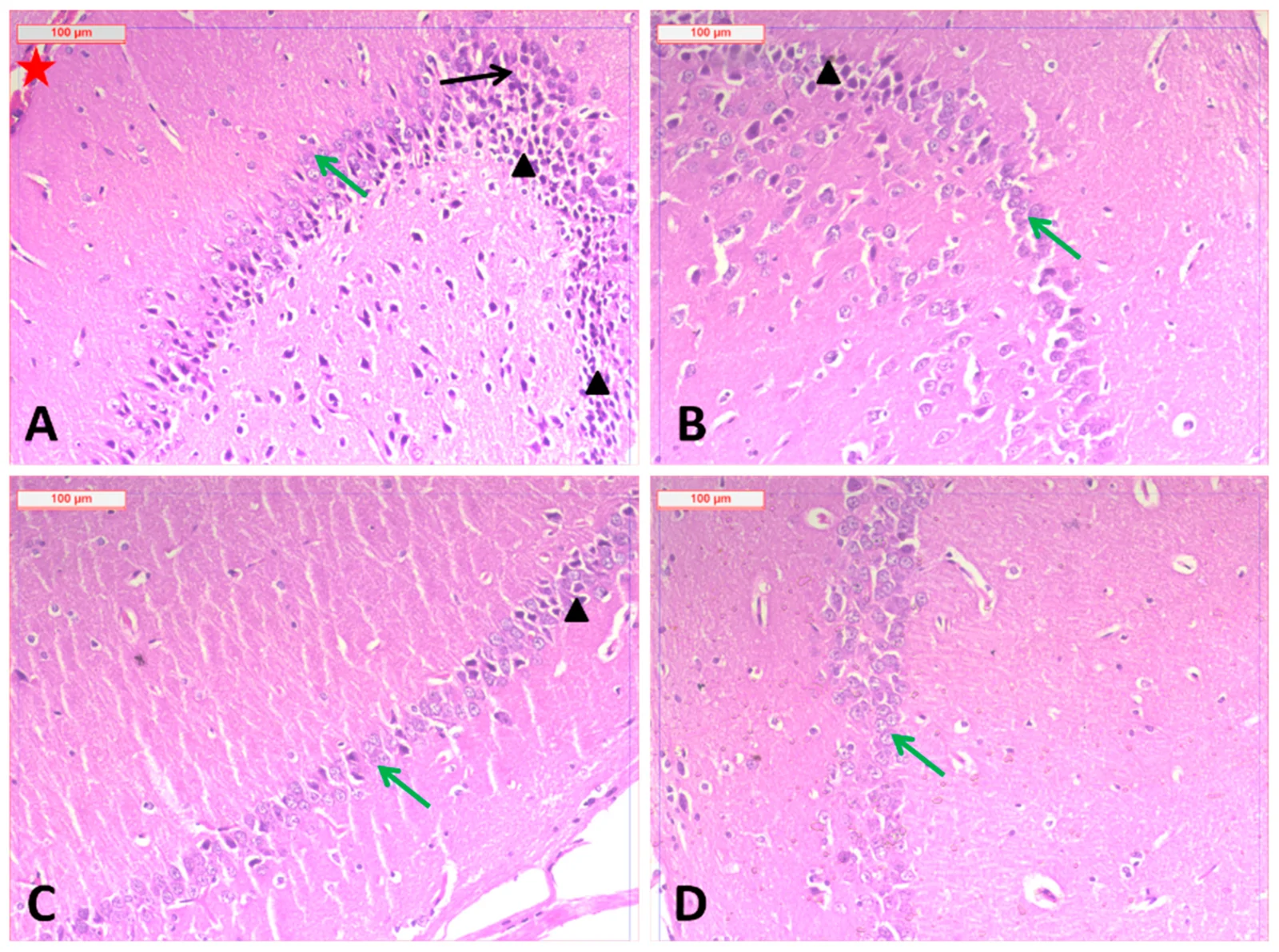
Photomicrographs of hippocampus region (**A**): vehicle group. (**B**): powder form PTPF. (**C**): nanoemulsion low-dose PTNL. (**D**): nanoemulsion high-dose PTNH. Black arrow: degenerated neurocyte. Green arrow: normal neurons. Red star: dilated blood vessel. Arrowhead: scattered apoptotic neurons (H&E, ×200).

Treatment with vehicle form revealed distorted and decreased thickness of the pyramidal cell layer, scattered apoptotic neurons with minimal normal neurons (Figure 11A). Treatment with variable forms of pterostilbene revealed variable degrees of improvement as follows: Treatment with powder form revealed marked improvement with normal neurons, a moderate number of apoptotic neurons and dilated blood vessels (Figure 11B). Treatment with low-dose nanoemulsion revealed marked improvement with normal neurons and a mild number of apoptotic neurons (Figure 11C). Treatment with high-dose nanoemulsion revealed marked improvement with few numbers of apoptotic neurons (Figure 11D).

**Figure 12:**
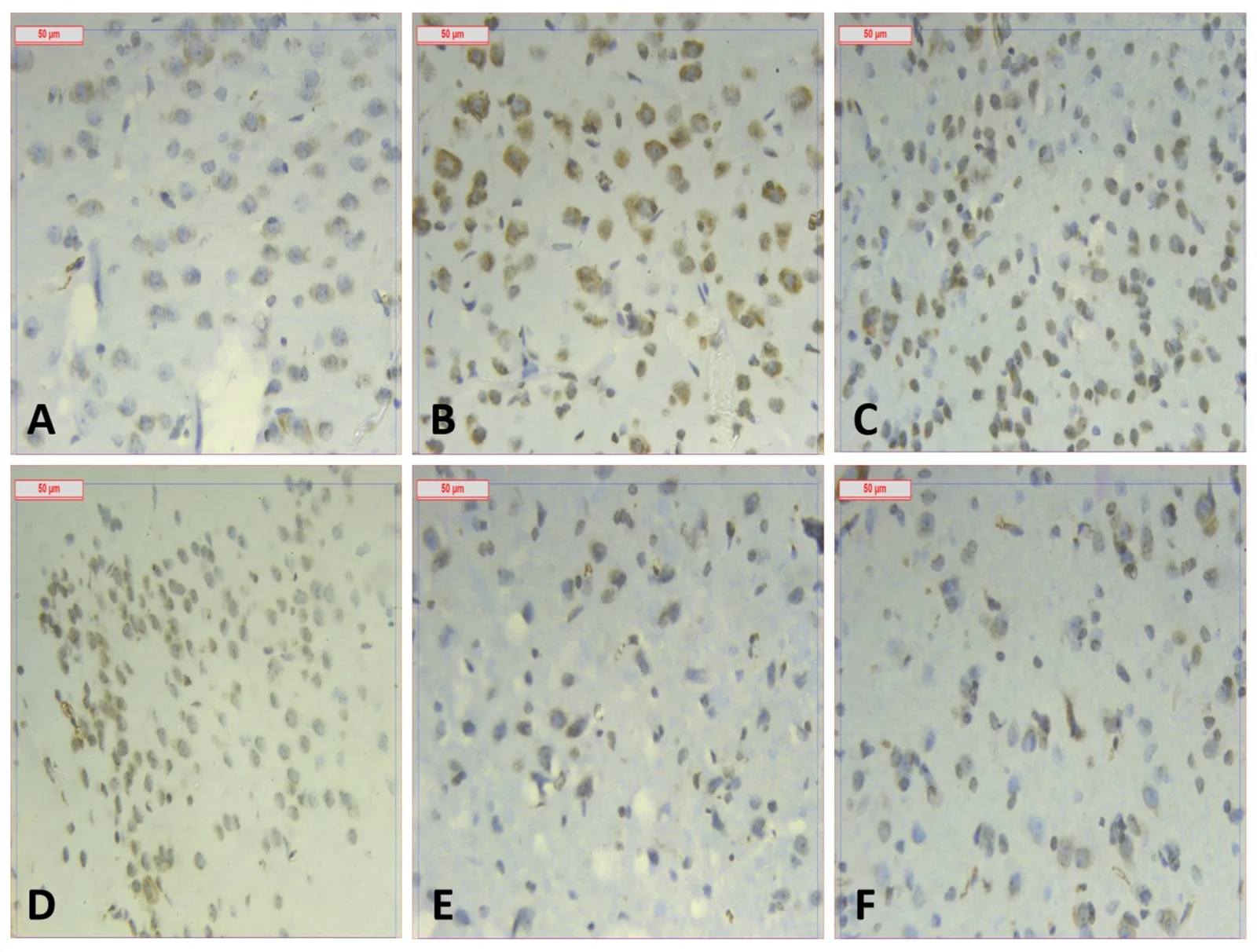
Photomicrographs of GFAP immunohistochemical expression in cortical region (**A**): Normal (**B**): Cyclophosphamide-treated group. (**C**): Vehicle group powder form. (**D**): Powder form (**E**): Nanoemulsion low-dose. (**F**): Nanoemulsion high-dose. (GFAP, ×400).

**Figure 13:**
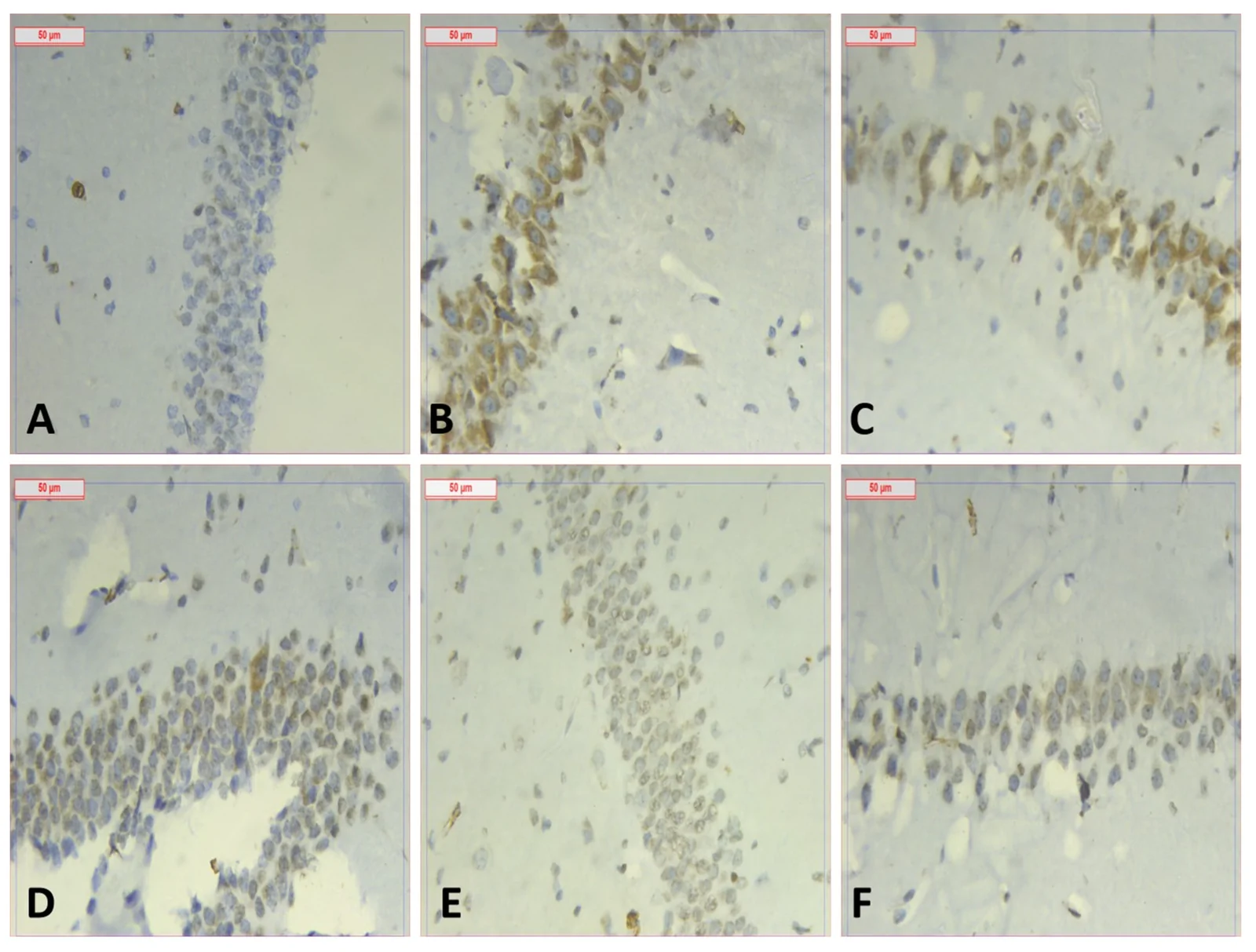
Photomicrographs of GFAP immunohistochemical expression in the hippocampus region (**A**): Normal (**B**): Cyclophosphamide-treated group. (**C**): Vehicle group powder form. (**D**): Powder form (**E**): Nanoemulsion low-dose. (**F**): Nanoemulsion high-dose. (GFAP, ×400).

Immunohistochemical expression of GFAP had various expressions in different groups; it had high positive expression in both of cyclophosphamide treated group and vehicle treated group, nearly negative in normal control group and in the high-dose nanoemulsion-treated group, while it had moderate expression in the low-dose nanoemulsion group and the powder-form-treated group (Figure 12 and Figure 13).

**Figure 14:**
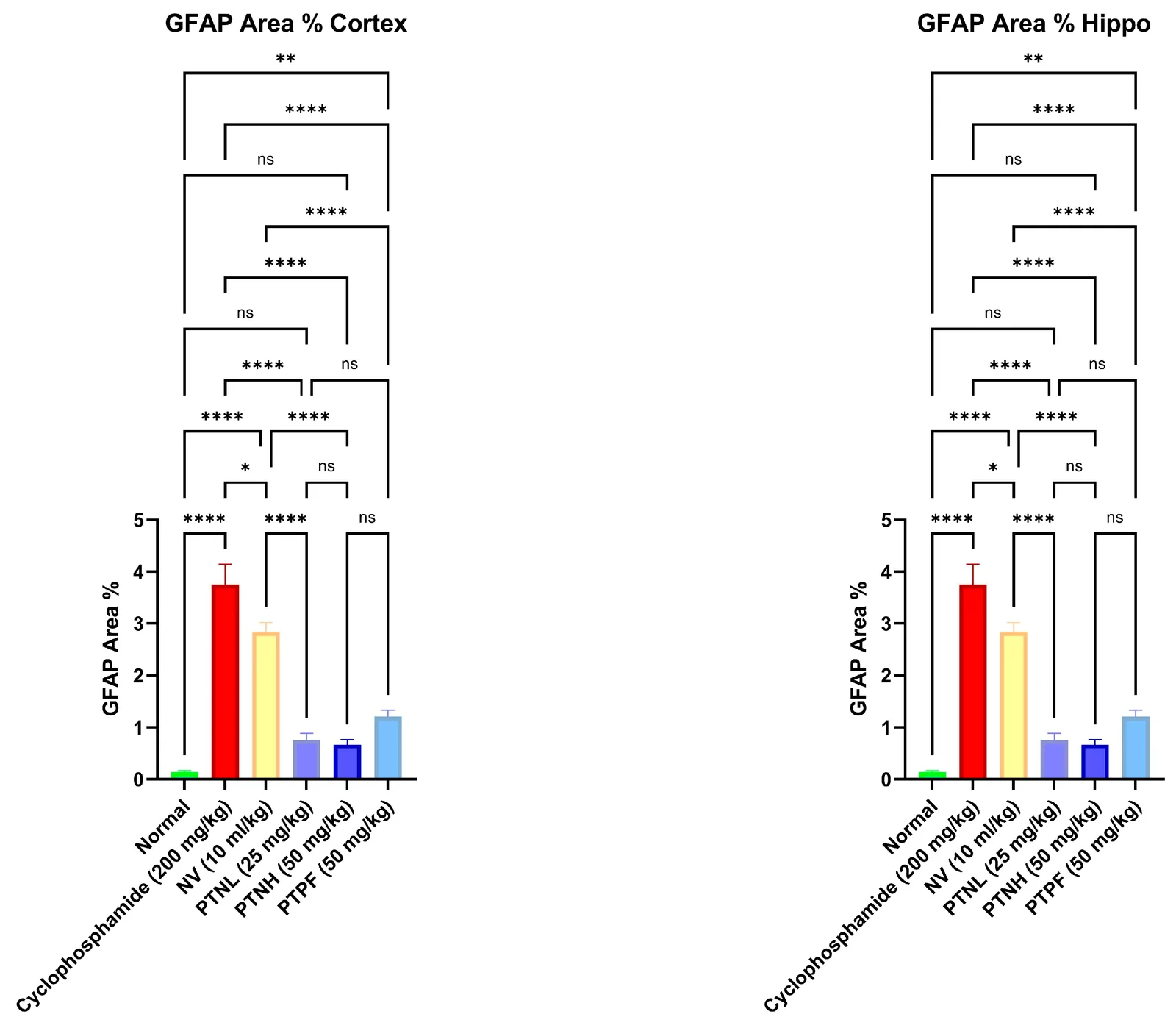
Variance of GFAP area% in different groups. Differences among groups were assessed by one-way analysis of variance (ANOVA), with Tukey–Kramer’s post hoc test. Results represent mean ± S.E (n = 8). Asterisks *, **, **** represent degree of significant difference, ns: non significant. NV: vehicle control; PTNL: low-dose pterostibene nanoemulsion group; PTNH: high-dose pterostibene nanoemulsion group; PTPF: pterostilbene freshly prepared powder suspension group.

Quantitative examination of the brown colored GFAP’s occupied area was measured at ×100 magnification. In the cortical region, GFAP area % was significantly elevated in the cyclophosphamide-treated group as compared to the normal control group (*p* < 0.0001). Regarding the area expression for the vehicle group, a slight significant difference was detected from the cyclophosphamide group (*p* = 0.0218). On the other hand, treatment with the powder form of pterostilbene revealed a moderate decrease in the area expression of GFAP, which was statistically different when compared with the cyclophosphamide group (*p* < 0.0001). Both nanoemulsion pterostilbene-treated groups, either low dose or high dose, had no significant difference from the normal control group (Figure 12 and Figure 14). Also, in the hippocampus region, GFAP area % had a great value in cyclophosphamide treated group, as confirmed by the presence of a significant difference between it and the normal control group (*p* < 0.0001). Meanwhile, the vehicle-treated group exhibited a minor yet significant reduction in the area expression as compared to the cyclophosphamide group (*p* = 0.0218). Treatment with the powder form of pterostilbene revealed a significant decrease in the area expression when compared to the cyclophosphamide control (*p* < 0.0001). Furthermore, both nanoemulsion pterostilbene-treated groups had low expression of GFAP, which appeared as decreased area% expression in both, with no significant difference from the normal control group (Figure 13 and Figure 14).

## 4. Discussion

Cyclophosphamide is one of the most used chemotherapeutic medications in several cancer types, such as ovarian and breast cancer [30,31], yet cyclophosphamide-induced central nervous system neuroinflammation and oxidative damage in neuronal cells are regarded as common and hazardous side effects that could hinder its application [32,33,34].

Brain tissue is specifically susceptible to oxidative damage owing to its low antioxidant capacity and elevated energy demand [32]. Cyclophosphamide in particular has been previously reported to be implicated in the disruption of cortical and hippocampus functions. accompanied by impaired neurogenesis, cerebral vascular alterations, and inflammatory and oxidative stress responses [7,31,35]. The current work aimed to clarify possible physiological mechanisms that underlie the cyclophosphamide-induced cortical and hippocampal toxicity, focusing mainly on the intracellular processes and emphasizing the protective function of pterostilbene by modifying pathways linked to oxidative stress, inflammation, and apoptosis.

Oxidative stress and inflammation are generally considered codependent [36]. Nuclear factor erythroid 2-related factor 2 (Nrf2) is principally known as the central transcription regulator combating oxidative stress, tissue injury and inflammation, with reported ability to stimulate the expression of the antioxidant enzyme heme oxygenase-1 (HO-1) [37,38,39]. Massive production of ROS was associated with downregulation of the antioxidant defense mechanism Nrf2/HO-1 cascade [40]. In addition, the nucleotide-binding oligomerization domain (NOD)-like receptor protein 3 (NLRP3) inflammasome has been shown to be linked to pro-inflammatory cytokines like cyclooxygenase 2 (COX-2) [36,38,41]. Apoptosis-associated speck-like protein (ASC) binds NLRP3 to pro-caspase-1 in an NLRP3 intracellular inflammasome complex, which is associated with caspase-1 activation and transforms the pro-inflammatory cytokine pro-IL-1β (associated with neural dysfunction) into its active secreted form. NLRP3 is considered one of the main components of the innate immune system [42,43]. On the other hand, both Nrf2 and HO-1 were demonstrated to have an inhibitory effect on NLRP3 activation [39,44]. Moreover, it was formerly stated that Nrf2 has anti-apoptotic functions by suppressing the pro-apoptotic Bax and upregulating the anti-apoptotic protein BCL2 [40].

The peroxisome proliferator activated receptor gamma (PPAR*γ*), which is known to regulate cell proliferation and differentiation [45], has been reported to induce anti-inflammatory responses through preventing pro-inflammatory gene expression and counteracting NLRP3 [46,47,48]. Additionally, PPARγ activation can directly promote the development of antioxidant enzymes and inhibit the production of ROS dependent on NADPH oxidase. In fact, it was demonstrated that activation of PPAR*γ* was associated with the activation of NRF2/HO-1 antioxidant pathway [48,49,50]. Meanwhile, the glial fibrillary acidic protein (GFAP) is the prominent monomeric intermediate filament protein in astrocytes, which are the chief type of glial cells in the central nervous system, and its increased expression usually represents the existence of neural injury or degeneration [51,52].

Correlational studies conducted on our results exposed the existence of an inverse correlation between PPAR-γ and NLPR3, in addition to a positive correlation between PPAR-γ and both Nrf2 and HO-1. Likewise, there was a positive correlation between Nrf2 and HO-1 as well as BCL2, along with an inverse correlation between Nrf2 and Bax. In addition, there was a negative correlation between Nrf2 and NLRP3 as well as between HO-1 and NLRP3. Meanwhile there was a positive correlation between COX-2 and NLRP3, in addition to a positive correlation between NLRP3 and IL-1β.

In the present study, PPAR-γ levels significantly decreased after cyclophosphamide treatment. In addition to its well-established anti-inflammatory, antioxidant, and neuroprotective functions, PPAR-γ has been shown to negatively influence the activation of the NLRP3 inflammasome and COX-2, as well as IL-1β production, and activate the Nrf2/HO-1 pathway. The cyclophosphamide-induced increased inflammatory response is probably exacerbated by the observed reduction in PPAR-γ. In addition, as mentioned above, Nrf2 could hinder the propagation of apoptosis by conquering Bax and enhancing BCL2 production. Consequently, disruption in the Nrf2/HO-1 pathway directly influences the apoptotic process. Our findings revealed that cyclophosphamide caused a significant decrease in both the cortical and the hippocampal levels of Nrf2, HO-1, PPAR-γ, as well as a significant increase in COX-2, IL-1β, NLRP3. Another important aftereffect of inflammation and oxidative stress is apoptotic dysregulation. As demonstrated by decreased BCL2 levels, elevated expression of Bax, and a significantly lower BCL2/Bax ratio in both the cortex and the hippocampus. Cyclophosphamide tipped the apoptotic balance in favor of cell death in the present investigation. Furthermore, histopathological examination revealed the existence of inflammation, blood vessel abnormalities and neuronal degeneration. Finally, immunohistochemical study confirmed an increased expression of glial fibrillary acidic protein (GFAP), the prominent monomeric intermediate filament protein in astrocytes, reflecting significant astrocytic alteration and neuroinflammation when comparing it to the normal control group and correlating with neural injury and degeneration.

Our results concurred with previously published reports confirming the neurotoxic effects of cyclophosphamide, represented by the increased NLPR3 expression and elevation in the IL-1β level [8,43]. Furthermore, cyclophosphamide resulted in downregulation of Nrf2 along with the upregulation of IL-1β and overexpression of Bax [31], and was also found to induce an imbalance of proteins linked to apoptosis, including the anti-apoptotic protein BCL2 and the pro-apoptotic protein Bax [53]. Another study highlighted that administration of cyclophosphamide in rats led to a significant increase in oxidative stress markers, disrupted antioxidant enzyme activities, upregulated inflammatory mediators, increased Bax and decreased BCL2. Moreover, histopathological examinations and immunohistochemical studies demonstrated neuronal degeneration, vascular hyperemia, and increased GFAP expression in brain tissue [54]. Similar findings were also formerly reported where cyclophosphamide was linked to the activation of astrocytes that was manifested in the form of increased expression of GFAP [55].

A potential cerebral neuroprotective role was denoted for pterostilbene, with superiority compared to resveratrol owing to its methoxy group, which increases its lipid solubility, thus facilitating its passage through the blood–brain barrier (BBB). The mechanism involved its ability to alleviate oxidative stress and inflammation as well as to regulate neurogenesis [15,56,57,58,59]. Several other reports involving different types of brain injuries delineated that pterostilbene was associated with activation of the Nrf2 signaling pathway, elevated HO-1, suppressed IL-1β, decreased COX-2 [60,61,62,63,64,65], inhibited NLRP3 [56,66,67] and upregulated BCL2, downregulated Bax, increased BCL2/Bax ratio and decreased GFAP expression [16,68]. Moreover, it was previously demonstrated that in a rat model of cerebral ischemia/reperfusion injury (CIRI), pterostilbene therapy inhibited cellular swelling and disintegration, macrophage infiltration, monocyte infiltration, and polymorphonuclear leucocyte degranulation [64]. Similar results were also observed in a CIRI model in mice, where pterostilbene improved the damage to brain tissue cell structure and improved cell arrangement in the hippocampal area [69].

The current study revealed that pterostilbene administration, either in its conventional powder form or the nanoemulsion form, managed to counteract the cyclophosphamide-induced intoxication features, via disrupting the cyclophosphamide-induced oxidative stress–inflammasome–cytokine axis through upregulating cortical and hippocampal PPAR-γ, Nrf2, HO-1, BCL2 levels and BCL2/Bax ratio in addition to downregulating NLRP3, IL-1β, COX-2, Bax and GFAP levels. The favorable performance of the nanoemulsion formulation, particularly when administered at the high dose level, emphasizes the significance of solubilization of pterostilbene to enhance its therapeutic potential, which was further supported by the histopathological and immunohistochemical assessment, evidenced by improved neuronal morphology, reduced degeneration and apoptosis, restoration of cortical and hippocampal architecture, and normalization of GFAP expression levels approaching those of the control group. These results collectively show that oxidative stress, neuroinflammation, inflammasome activation, and apoptosis are dynamically associated with cyclophosphamide-induced neurotoxicity. Activating the NRF2/HO-1 antioxidant pathway, decreasing COX-2 and IL-1β-mediated inflammation, reducing NLRP3 inflammasome activation, re-establishing PPAR-γ signaling, and apoptotic equilibrium were all associated with the neuroprotective effects of pterostilbene, particularly when its solubilization was attempted through nanoformulation, resulting in greater neuroprotection.

It is worth mentioning that although the group ingesting the nano-vehicle unloaded with pterostilbene showed a slight improvement in some parameters, in most cases the overall effect was mostly non-significantly different from the cyclophosphamide control.

A limitation of the present study is that groups receiving pterostilbene alone or unloaded nanoemulsion alone in healthy animals were not included. Because the study was specifically designed to investigate protection against cyclophosphamide-induced toxicity while minimizing animal use in accordance with ethical principles, the experimental design was limited to groups relevant to this objective. Consequently, the independent effects of pterostilbene and the nanoemulsion vehicle under normal physiological conditions could not be fully evaluated. As the unloaded nanoemulsion showed modest effects on some measured parameters, the observed treatment effects should therefore be interpreted with appropriate caution. Future studies incorporating these additional control groups would help clarify the independent contributions of pterostilbene and the nanoemulsion vehicle. Moreover, pH evaluation and in vitro drug release studies were not performed within the scope of the present work, and should be included in future studies to provide a full picture of formulation stability and pterostilbene release behavior.

## 5. Conclusions

The clinical efficacy of cyclophosphamide, a commonly used chemotherapeutic drug, is frequently restricted by off-target neurotoxicity, which is primarily caused by oxidative stress, neuroinflammation, and apoptosis. The current investigation confirmed the harmful effects of cyclophosphamide on brain tissue, inducing significant biochemical and molecular changes in the hippocampus and cerebral cortex. Suppression of antioxidant defense pathways, stimulation of inflammatory and inflammasome signaling, and disruption of apoptotic balance were all indicators of these changes. Pterostilbene treatment, especially in its nanoemulsion, significantly reduced these degenerative alterations, underscoring its strong neuroprotective properties.

## Data Availability

The original contributions presented in this study are included in the article/Supplementary Materials. Further inquiries can be directed to the corresponding author.

## Supplementary Materials

The following supporting information can be downloaded at: https://www.mdpi.com/article/10.3390/biomedicines14081779/s1, Supplementary Figure S1: TEM micrograph of pterostilbene nanoemulsion (50 mg/L) concentration.

## Abbreviations

The following abbreviations are used in this manuscript:

ASC	Apoptosis-associated speck-like protein
Bax	BCL2-associated X
BBB	Blood–brain barrier
BCL2	B-cell lymphoma 2
Nrf2	Nuclear erythroid-related factor
CIRI	Cerebral Ischemia/Reperfusion Injury
COX-2	Cyclooxygenase 2
GFAP	Glial cell marker glial fibrillary acidic protein
H&E	Hematoxylin and Eosin
HO-1	Heme oxygenase-1
NADPH	Nicotinamide Adenine Dinucleotide Phosphate
NLRP3	Nucleotide-binding oligomerization domain (NOD)-like receptor protein 3 inflammasome
IL-1β	Interleukin-1 beta
PPAR- γ	Peroxisome proliferator-activated receptor gamma
ROS	Reactive oxygen species
