# Supplementary material for: Pterostilbene Combating Cyclophosphamide-Induced Central Neuro-Inflammation in Rats: Effect on GFAP, PPAR- γ, NLRP3, COX-2 and Nrf2/HO-1 Pathway
Source: Biomedicines. 2026 Aug 7;14(8):1779. doi: 10.3390/biomedicines14081779 (PMC13509188; doi:10.3390/biomedicines14081779)
Supplement: Supplementary file 1 [file biomedicines-14-01779-s001.zip › biomedicines-4454870-supplementary.pdf]

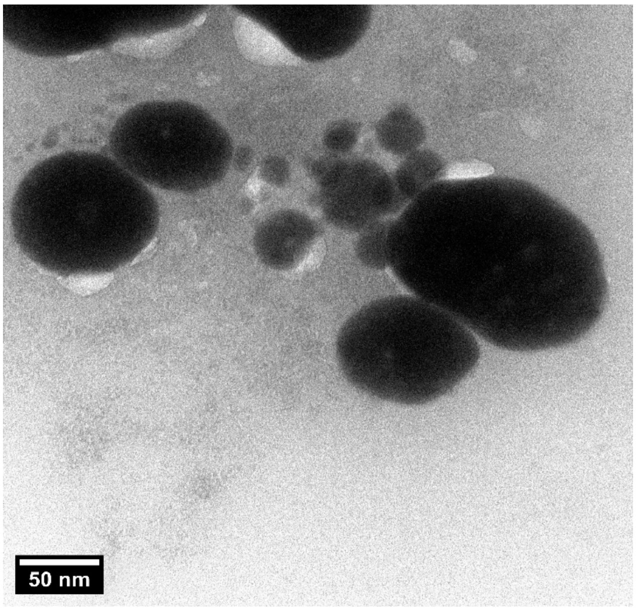

Supplementary Figure S1: TEM micrograph of pterostilbene nanoemulsion (50 mg/L) concentration.
